# Differential regulations of vestibulo-ocular reflex and optokinetic response by β- and α2-adrenergic receptors in the cerebellar flocculus

**DOI:** 10.1038/s41598-017-04273-9

**Published:** 2017-06-21

**Authors:** Ryo Wakita, Soshi Tanabe, Kazunari Tabei, Asako Funaki, Takuma Inoshita, Tomoo Hirano

**Affiliations:** Department of Biophysics, Graduate School of Science, Kyoto University, Sakyo-ku, Kyoto 606-8502 Japan

## Abstract

Norepinephrine modulates synaptic plasticity in various brain regions and is implicated in memory formation, consolidation and retrieval. The cerebellum is involved in motor learning, and adaptations of the vestibulo-ocular reflex (VOR) and optokinetic response (OKR) have been studied as models of cerebellum-dependent motor learning. Previous studies showed the involvement of adrenergic systems in the regulation of VOR, OKR and cerebellar synaptic functions. Here, we show differential contributions of β- and α2-adrenergic receptors in the mouse cerebellar flocculus to VOR and OKR control. Effects of application of β- or α2-adrenergic agonist or antagonist into the flocculus suggest that the β-adrenergic receptor activity maintains the VOR gain at high levels and contributes to adaptation of OKR, and that α2-adrenergic receptor counteracts the β-receptor activity in VOR and OKR control. We also examined effects of norepinephrine application, and the results suggest that norepinephrine regulates VOR and OKR through β-adrenergic receptor at low concentrations and through α2-receptor at high concentrations.

## Introduction

The cerebellum is involved in motor learning, and adaptations of the vestibulo-ocular reflex (VOR) and optokinetic response (OKR) have been studied as models of motor learning^1–4^. VOR and OKR are reflex eye movements that work to suppress the image motion on a retina during head movement. In VOR, turning the head stimulates the vestibular organ, resulting in turning of the eyeball in the direction opposite to the head turn. In OKR, movement of the large visual field (optokinetic stimulation) induces the eyeball to turn in the same direction as the image motion. VOR and OKR show adaptive changes if VOR or OKR cannot suppress the retinal slip during head motion or optokinetic stimulation. For example, if sinusoidal rotation of an animal is continuously coupled with sinusoidal rotation of a screen surrounding the animal in the opposite or same direction, the gain of VOR gradually increases or decreases, respectively^1, 2^. On the other hand, when the surrounding screen is rotated at a relatively high speed without animal rotation, the eyeball turn cannot follow the screen movement at first. However, if the optokinetic stimulation continues for a certain time, the speed of the eyeball turn gets faster such that it can follow the screen movement better^3, 4^. These phenomena are adaptations of VOR and OKR, which work to improve stabilization of the visual image on the retina during animal movement.

Both VOR and OKR are mediated by brainstem nuclei including vestibular nuclei, and regulated by Purkinje cell outputs from the cerebellar flocculus. In VOR, the head movement is sensed by vestibular organs, which send outputs to vestibular nuclei and to the flocculus. In OKR the retinal slip information is sent to the nucleus of optic tract and the accessory optic system, which send outputs to the nucleus reticularis tegmenti pontis (NRTP) and the inferior olive nuclei (ION). The NRTP sends outputs to the vestibular nuclei and to the flocculus. In horizontal OKR or VOR, neurons in the vestibular nuclei send outputs to neurons in the oculomotor or the abducens nuclei, which directly control extraocular muscles^5^. The retinal slip information from the ION, vestibular information, efference copy of motor command, etc., converge on Purkinje cells of the flocculus^1, 2, 6, 7^, and the inhibitory output signals of these Purkinje cells onto neurons in vestibular nuclei control VOR and OKR^1, 2^.

Synaptic plasticity mechanisms dependent on climbing fiber inputs from the ION in a Purkinje neuron such as long-term depression (LTD) at excitatory parallel fiber – Purkinje neuron synapses and rebound potentiation (RP) at inhibitory interneuron – Purkinje neuron synapses, and also synaptic plasticity in the vestibular nuclei, seem to contribute to adaptation of VOR and OKR^1–3, 8, 9^. Although the roles and regulatory neuronal pathways are similar between VOR and OKR, differential regulations of VOR and OKR by cerebellar synaptic plasticity mechanisms have been reported^8, 10, 11^. In addition, Faulstich *et al*.^12^ reported interesting one-directional interaction between them. Both gain-increase and gain-decrease VOR training increase the OKR gain similarly to OKR training, but OKR training does not affect VOR.

A previous study showed that administration of β-adrenergic agonist to the flocculus increases the OKR gain but not the VOR gain^13^. The norepinephrine input to the cerebellum activating adrenergic receptors comes from the locus coeruleus^14–16^. Emotional arousal activates locus coeruleus neurons, and the released norepinephrine modulates synaptic plasticity in various brain regions and is implicated in memory formation, consolidation and retrieval^16, 17^. It has also been proposed that norepinephrine plays a role in modulation of cerebellum-dependent learning^18, 19^, and injection of β-adrenergic antagonist into the rabbit flocculus diminishes the gain-up adaptation of VOR^20, 21^. However, it is not known whether the β-adrenergic receptor contributes to the gain increase adaptation of OKR induced by various training paradigms. It is also enigmatic how the α2-adrenergic receptor, which can counteract activity of the β-adrenergic receptor^22^, influences VOR and OKR. In addition, it is unclear which adrenergic receptor (β or α2) is predominantly activated by norepinephrine in the flocculus. We have addressed these questions and attempted to clarify the respective roles of β- and α2-adrenergic receptors in regulation and adaptations of VOR and OKR in mice.

## Results

### Effects of β-adrenergic agonist or antagonist on VOR and OKR

**Figure 1:**
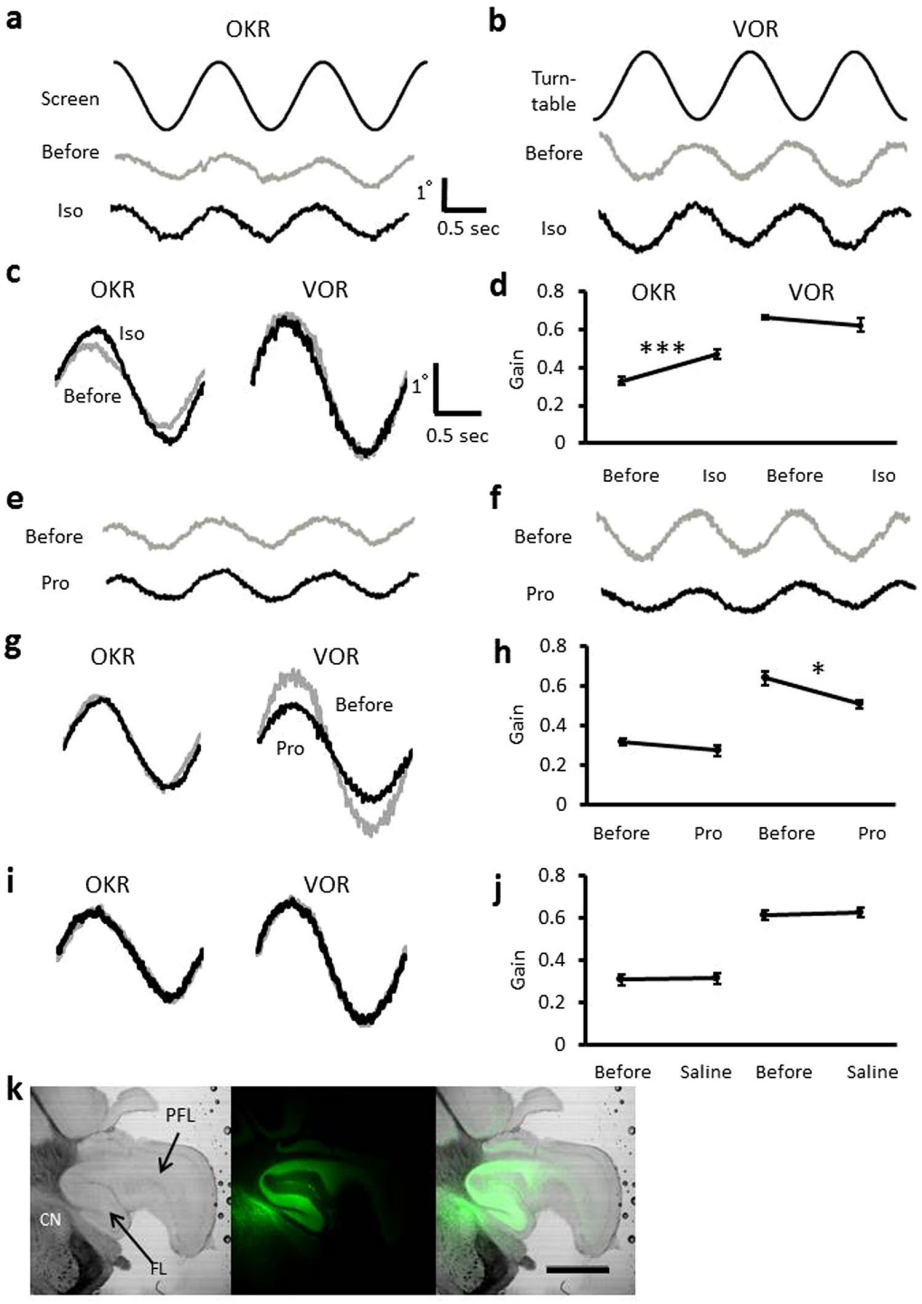
Effects of β-adrenergic agonist isoproterenol or antagonist propranolol on OKR and VOR. (**a**,**b**), Representative eye position traces during OKR or VOR before and after administration of isoproterenol (Iso). The command signal corresponding to the position of screen (**a**) or turntable (**b**) is also presented (top). (**c**) Averaged (10 cycles) traces of eye position before (grey) and after (black) isoproterenol administration (**d**) Gain changes of OKR and VOR by isoproterenol administration. (**e,f**) Eye position traces during OKR (**e**) and VOR (**f**) before and after administration of propranolol. (**g**) Averaged eye position traces. (**h**) Gain changes of OKR and VOR caused by propranolol administration. (**i**) Averaged traces of eye position before and after saline administration. (**j**) Gains of OKR and VOR before and after administration of saline. Error bars indicate SEM. *p < 0.05, ***p < 0.001, paired Student’s t-test. (**k**) Photographs of the flocculus injected with acriflavine (green). FL, flocculus; PFL, para-flocculus; CN, cochlear nucleus. Scale bar indicates 1 mm.

First, we examined the effects of β-adrenergic agonist isoproterenol and antagonist propranolol on OKR and VOR. Administration of isoproterenol into the left flocculus increased the OKR gain measured in the right eye from 0.33 ± 0.02 to 0.47 ± 0.02 (n = 6; p < 0.001, paired Student’s t-test), but did not affect the VOR gain (before, 0.66 ± 0.01; after, 0.63 ± 0.04; n = 6; p = 0.42) (Fig. 1a–d) in accord with a previous report^13^. In contrast, application of β-adrenergic antagonist propranolol did not affect the OKR gain (before, 0.32 ± 0.02; after, 0.28 ± 0.03; n = 6; p = 0.11), but decreased the VOR gain from 0.64 ± 0.03 to 0.51 ± 0.02 (n = 6; p = 0.017) (Fig. 1e–h). Administration of saline affected neither the OKR gain (before, 0.31 ± 0.02; after, 0.31 ± 0.03; n = 6; p = 0.81) nor the VOR gain (before, 0.61 ± 0.02; after, 0.63 ± 0.02; n = 6; p = 0.56) (Fig. 1i,j).

In some cases, after the eye movement recording, the drug application sites were examined in the histological preparations to detect fluorescent signals of either lucifer yellow or acriflavine hydrochloride that had been applied together with the drug. We detected fluorescent signals in the flocculus, and in addition the signal spread to the para-flocculus and/or cochlear nuclei in most cases (Fig. 1k).

**Figure 2:**
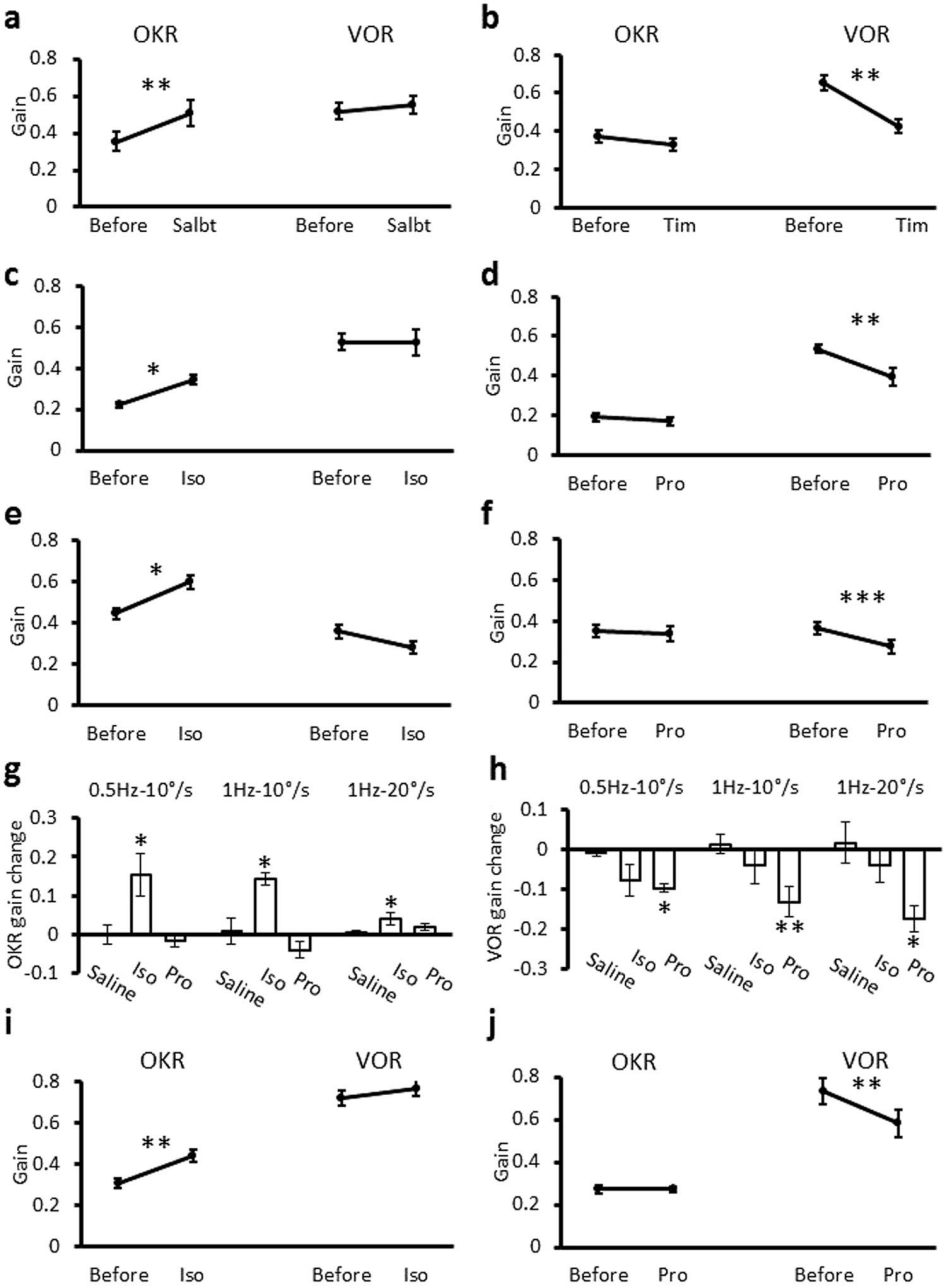
OKR and VOR gain changes induced by β-adrenergic agonist or antagonist in various conditions. (**a**,**b**) OKR and VOR gain changes induced by contralateral injection of β-adrenergic agonist salbutamol (**a**) or antagonist timolol (**b**). (**c**,**d**) Effects of contralateral injection of isoproterenol (**c**) or propranolol (**d**) on gains of OKR and VOR during sinusoidal rotation of the screen or the turntable at 1 Hz, 20°/sec peak velocity. (**e**,**f**) Effects of isoproterenol (**e**) or propranolol (**f**) injection on gains of OKR and VOR during sinusoidal rotation of the screen or the turntable at 0.5 Hz, 10°/sec peak velocity. *p < 0.05, **p < 0.01, ***p < 0.001, paired Student’s t-test. (**g**,**h**) Gain changes of OKR (**g**) and VOR (**h**) induced by administration of saline, isoproterenol or propranolol under different rotation conditions. *p < 0.05, **p < 0.01, compared with saline-injected control, Dunnett’s test. (**i**,**j**) OKR and VOR gain changes induced by ipsilateral injection of isoproterenol (**i**) or propranolol (**j**). **p < 0.01, paired Student’s t-test. Error bars indicate SEM.

We also examined the effects of another β-adrenergic agonist (salbutamol) and antagonist (timolol). Salbutamol increased the OKR, but did not affect the VOR gain (Fig. 2a, Supplementary Tables S1, S2), similarly to isoproterenol. Timolol did not affect the OKR gain, but decreased the VOR gain similarly to propranolol (Fig. 2b, Supplementary Tables S1, S2).

Next, we examined the effects of isoproterenol and propranolol on OKR and VOR in different optokinetic or vestibular stimulation conditions (1 Hz, 20°/sec peak velocity; 0.5 Hz, 10°/sec peak velocity). The effects on gains were qualitatively similar to those obtained with 1 Hz, 10°/sec peak velocity stimulation (Fig. 2c–h; Supplementary Tables S3, S4).

Ipsilateral injection to the right flocculus of isoproterenol or propranolol was also performed, and the effects on OKR and VOR were examined. These effects were qualitatively similar to those of contralateral injection (Fig. 2i,j; Supplementary Tables S1, S2). The results together indicate that β-adrenergic receptor activation enhances OKR but not VOR, and suggest that in the flocculus of a naive mouse that has not experienced drug application or training, endogenous β-adrenergic agonist, presumably norepinephrine, is present at a certain concentration and supports the VOR gain.

We also measured the phase of OKR or VOR in each condition (Supplementary Tables S5–S8). In general, β-adrenergic agonist tended to decrease the phase delay of OKR and increase the phase lead of VOR. However, some results were not statistically significant, and some effects, such as that of isoproterenol on the OKR phase, were not consistent among different conditions (Supplementary Table S7). Therefore, in this study we concentrated on analyses of gains of OKR and VOR.

**Figure 3:**
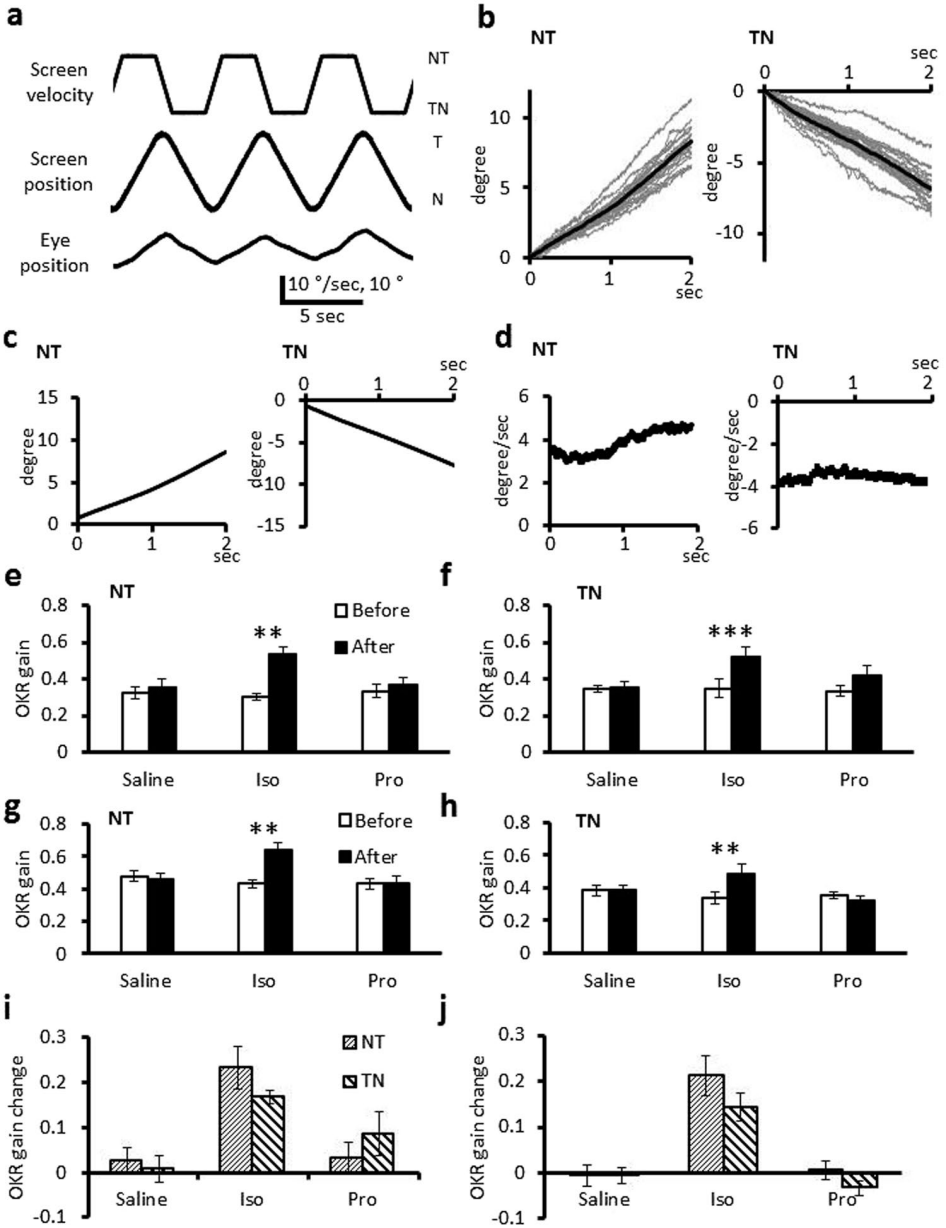
NT and TN eye movements during constant-velocity optokinetic stimulation. (**a**) The command velocity signal for the screen movement, screen position and a representative corresponding eye position. (**b**) Eye position traces during constant-velocity optokinetic stimulation in both NT and TN directions recorded from a mouse before administration of a drug. Gray lines indicate each data and black lines indicate averaged traces from a mouse. (**c,d**) Mean eye position (**c**) and velocity (**d**) traces obtained from 18 mice. Moving average for 0.1 sec was applied for velocity traces. (**e–h**) Effects of administration of isoproterenol, propranolol or saline on the eye velocity during the former (**e,f**) or the latter (**g,h**) 1 sec of 2 sec constant-velocity optokinetic stimulation in NT (**e,g**) or TN (**f,h**) direction. (**i,j**) Effects of administration of isoproterenol, propranolol or saline on the eye velocity changes during the former (**i**) or latter (**j**) 1 sec of 2 sec constant-velocity optokinetic stimulation. Error bars indicate SEM. **p < 0.01, ***p < 0.001, paired Student’s t-test.

Recently asymmetrical regulation of eye movement during constant-velocity optokinetic stimulation was reported^23^. We examined effects of administration of isoproterenol or propranolol on the eye movement during constant-velocity rotation of the screen at 10 °/sec for 2 sec in both directions (Fig. 3a). Before administration of a drug or saline, the velocity of temporal-to-nasal (TN) eye movement did not change significantly during 2 sec constant-velocity screen movement, whereas the velocity of nasal-to-temporal (NT) movement tended to increase (Fig. 3b–d). Thus, the eye velocity of NT movement during the former 1 sec (0.32 ± 0.02, n = 18) was smaller than that during the latter 1 sec (0.45 ± 0.02, p < 0.001, paired t-test). On the other hand, they were similar (former, 0.35 ± 0.02; latter, 0.36 ± 0.02; n = 18, p = 0.422) in TN movement.

Administration of isoproterenol facilitated the eye movement during the former as well as the latter 1 sec period of constant-velocity optokinetic stimulation in both directions (former NT, 0.23 ± 0.05 increase, p = 0.004; TN, 0.17 ± 0.01 increase, p < 0.001; latter NT, 0.21 ± 0.04 increase, p = 0.004; TN, 0.14 ± 0.03 increase, p = 0.005; n = 6, paired t-test) (Fig. 3e–h, Supplementary Table S9). The velocity increase was not significantly different between NT and TN directions in both the former and latter periods (p = 0.185 and 0.216, Student’s t-test) (Fig. 3i,j, Supplementary Table S9). Application of propranolol or saline did not significantly affect the eye velocity during constant-velocity optokinetic stimulation in either direction (Fig. 3e–h, Supplementary Table S9). Thus, we did not detect significant asymmetrical effects of β-adrenergic agonist or antagonist on OKR during the constant-velocity optokinetic stimulation, although we found directional asymmetry of eye movement time course before application of a drug.

### Effects of β-adrenergic agonist or antagonist on VOR and OKR adaptation

**Figure 4:**
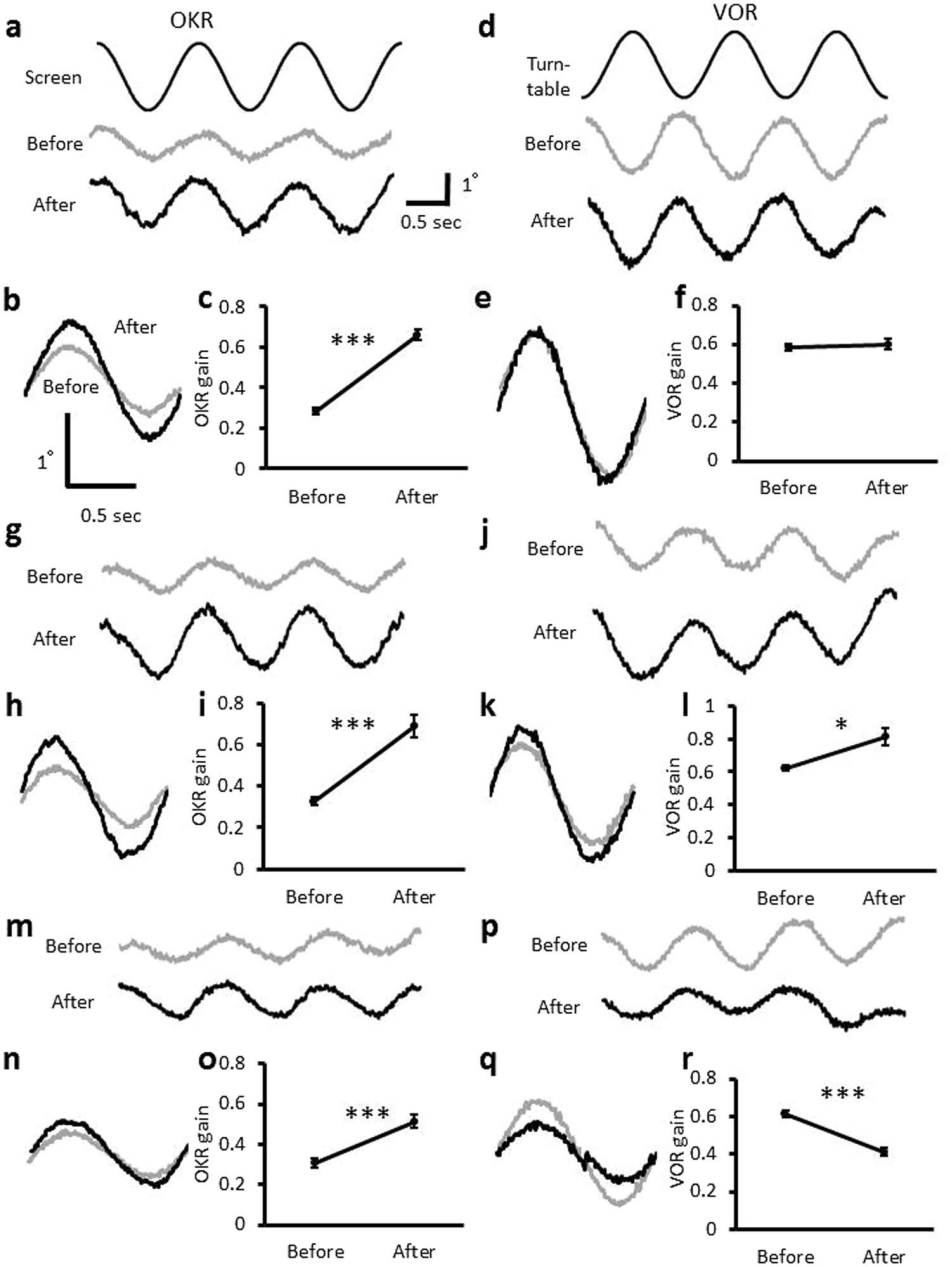
Effects of OKR, gain-up or gain-down VOR training. (**a**) Eye movement traces during OKR (**a**) before and after OKR training presented with the command signal corresponding to the screen position. (**b**) Averaged (10 cycles) traces of eye position during OKR before (grey) and after (black) OKR training. (**c**) OKR gain change by OKR training. (**d**) Eye movement traces during VOR before and after OKR training presented with the command signal corresponding to the turntable position. (**e**) Averaged traces of eye position during VOR. (**f**) VOR gain change by OKR training. (**g**) Eye movement traces during OKR before and after gain-up VOR training. (**h**) Averaged traces of eye position during OKR. (**i**) OKR gain change by gain-up VOR training. (**j**) Eye movement traces during VOR before and after gain-up VOR training. (**k**) Averaged traces of eye position during VOR. (**l**) VOR gain change by gain-up VOR training. (**m**) Eye movement traces during OKR before and after gain-down VOR training. (**n**) Averaged traces of eye position during OKR. (**o**) OKR gain change by gain-down VOR training. (**p**) Eye movement traces during VOR before and after gain-down VOR training. (**q**) Averaged traces of eye position during VOR. (**r**) VOR gain change by gain-down VOR training. Error bars indicate SEM. *p < 0.05, ***p < 0.001, paired Student’s t-test.

Previous studies showed that not only OKR training but also gain-up VOR or gain-down VOR training increased the OKR gain^12^. We confirmed that OKR training increased the OKR gain from 0.29 ± 0.02 to 0.66 ± 0.03 (0.38 ± 0.03 increase; n = 6; p < 0.001), but did not affect the VOR gain (before, 0.59 ± 0.02; after, 0.60 ± 0.03; n = 6; p = 0.64), and that gain-up VOR training increased not only the VOR gain (before, 0.62 ± 0.02; after, 0.81 ± 0.05; 0.19 ± 0.05 increase; n = 6; p = 0.011) but also the OKR gain (before, 0.33 ± 0.02; after, 0.69 ± 0.05; 0.36 ± 0.04 increase; n = 6; p < 0.001), and that gain-down VOR training decreased the VOR gain (before, 0.61 ± 0.02; after, 0.41 ± 0.02; 0.20 ± 0.02 decrease; n = 6; p < 0.001), but increased the OKR gain (before, 0.31 ± 0.02; after, 0.52 ± 0.03; 0.21 ± 0.03 increase; n = 6; p = 0.001) (Fig. 4; Supplementary Tables S1, S2).

The phase changes of OKR and VOR induced by OKR or VOR training were also measured (Supplementary Tables S5, S6). OKR training decreased the phase delay of OKR in several conditions. The VOR phase lead tended to be increased by OKR training or VOR gain-down training, although the effects of drugs on the VOR phase were unclear and difficult to interpret consistently.

**Figure 5:**
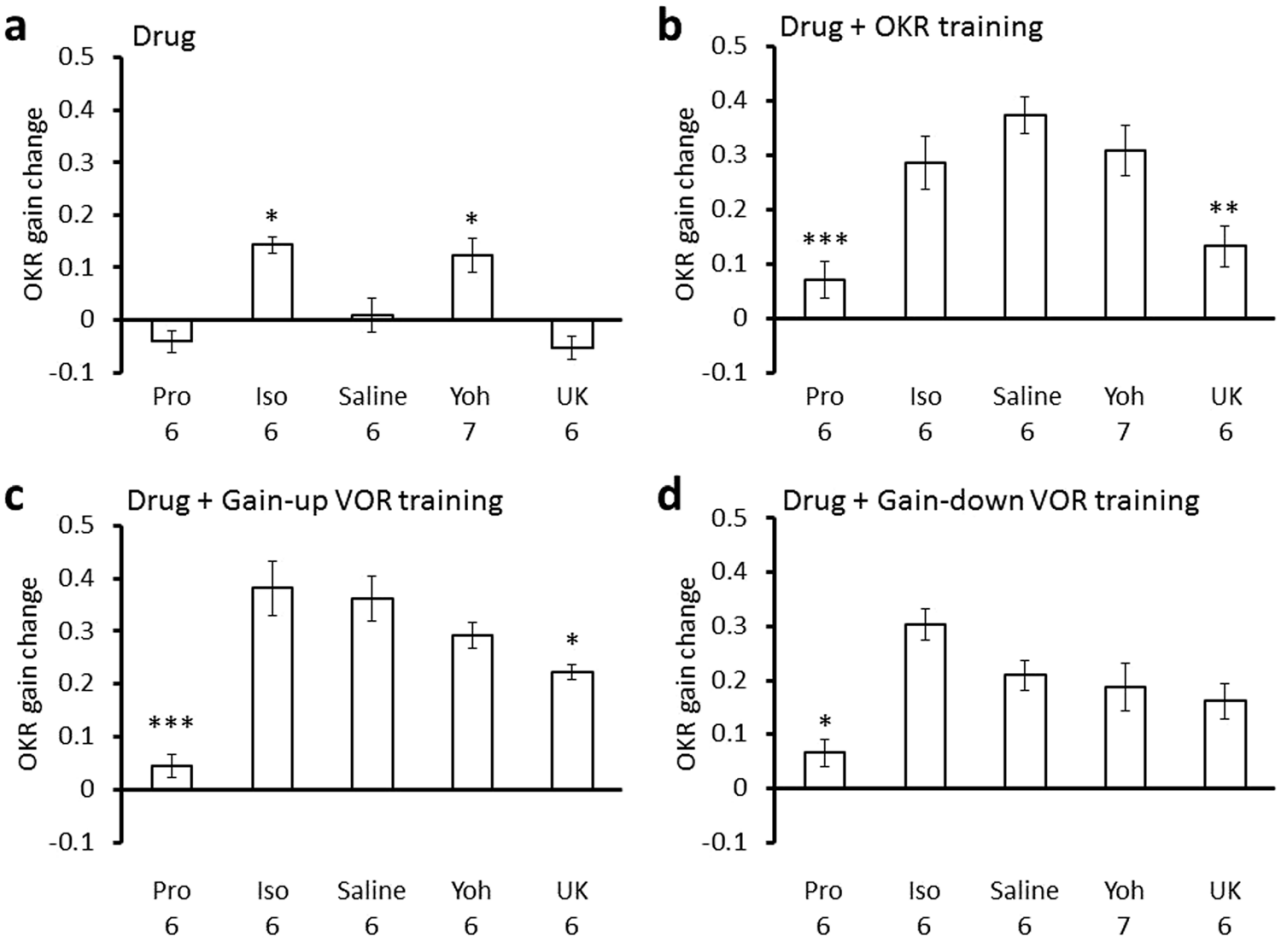
OKR gain changes induced by various treatments. (**a**) Effects of administration of propranolol (Pro), isoproterenol (Iso), saline, yohimbine (Yoh) or UK14304 (UK) on the OKR gain. Pro, Iso and Saline data are same as those shown in Fig. 2g. They are presented again for comparison. (**b–d**) Effects of administration of propranolol, isoproterenol, saline, yohimbine or UK14304 on the OKR gain increase induced by OKR training (**b**), by gain-up VOR training (**c**) or by gain-down VOR training (**d**). Numbers under each graph indicate numbers of mice, and error bars indicate SEM. *p < 0.05, **p < 0.01, ***p < 0.001, compared with saline-injected control, Dunnett’s test.

**Figure 6:**
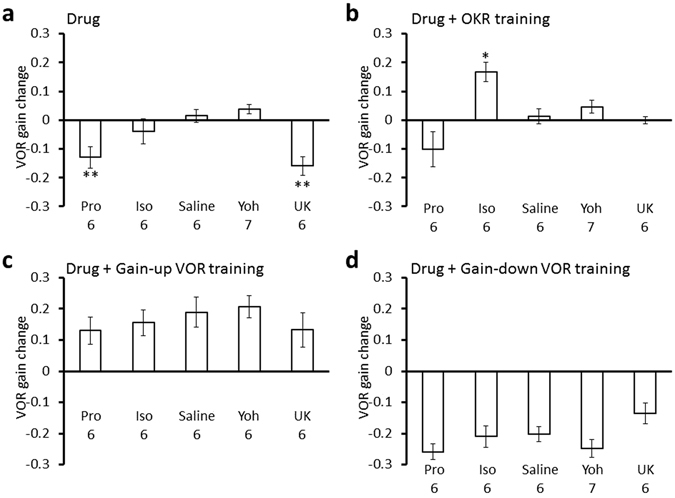
VOR gain changes induced by various treatments. (**a**) Effects of administration of propranolol (Pro), isoproterenol (Iso), saline, yohimbine (Yoh) or UK14304 (UK) on the VOR gain. Pro, Iso and Saline data are same as those shown in Fig. 2h. They are presented again for comparison. (**b–d**) Effects of administration of propranolol, isoproterenol, saline, yohimbine or UK14304 on the VOR gain changes induced by OKR training (**b**), by gain-up VOR training (**c**) or by gain-down VOR training (**d**). Numbers under each graph indicate numbers of mice, and error bars indicate SEM. *p < 0.05, **p < 0.01, compared with saline-injected control, Dunnett’s test.

Next, we examined effects of β-adrenergic agonist or antagonist on the OKR and VOR gain changes caused by trainings. Administration of propranolol significantly suppressed the gain increase of OKR induced by OKR training (0.07 ± 0.03; n = 6; p < 0.001 compared with the saline-injected control, 0.38 ± 0.03, Dunnett’s test), by gain-up VOR training (0.05 ± 0.02; n = 6; p < 0.001) or by gain-down VOR training (0.07 ± 0.03; n = 6; p = 0.012) (Fig. 5; Supplementary Table S1), whereas propranolol administration did not affect VOR gain changes induced by any training (Fig. 6, Supplementary Table S2). These results indicate that activation of β-adrenergic receptor is involved in the gain-increase change of OKR by various types of training.

In contrast, administration of β-adrenergic agonist isoproterenol did not affect changes of OKR gain or VOR gain caused by VOR training (Figs 5 and 6; Supplementary Tables S1, S2). However, isoproterenol increased the VOR gain after OKR training (before, 0.64 ± 0,04; after, 0.81 ± 0.03; 0.17 ± 0.03 increase; n = 6; p = 0.04, paired Student’s t-test) (Fig. 6; Supplementary Table S2). This result is surprising, as neither isoproterenol administration by itself nor OKR training without any drug affected the VOR gain (Supplementary Table S2). Coupling of the β−adrenergic receptor activation and OKR training seems to have enhanced the sensitivity of cerebellar flocculus neuronal pathways to the vestibular input by some unknown mechanism.

### Effects of α2-adrenergic agonist or antagonist on VOR and OKR

We next examined the involvement of α2-adrenergic receptor in the regulation of VOR and OKR. α2-adrenergic receptor is coupled to Gi protein and can counteract the activity of β-adrenergic receptor coupled to Gs protein^22, 24, 25^. Administration of α2-adrenergic agonist UK14304 did not affect the OKR gain, but decreased the VOR gain (before, 0.65 ± 0.04; after, 0.49 ± 0.04; n = 6; p = 0.004), similarly to application of β-adrenergic antagonist propranolol (Figs 5 and 6; Supplementary Tables S1, S2). On the other hand, α2-adrenergic antagonist yohimbine increased the OKR gain (before, 0.30 ± 0.02; after, 0.42 ± 0.03; n = 7; p = 0.010) without affecting the VOR gain, similarly to β-adrenergic agonist isoproterenol (Figs 5 and 6; Supplementary Tables S1, S2). We confirmed that DMSO, which was used to dissolve UK14304 affected neither OKR nor VOR (Supplementary Tables S1, S2). These results suggest that a certain level of cAMP concentration in some flocculus neurons might be necessary to maintain the gain of VOR at a high level, because β-adrenergic receptor increases intracellular cAMP concentration through Gs protein and α2-adrenergic receptor decreases it through Gi protein.

The effects of α2-adrenergic agonist or antagonist on the changes of OKR or VOR gain by OKR or VOR training were also examined (Figs 4 and 5, Supplementary Tables S1, S2). UK14304 suppressed the gain increase of OKR by OKR training (0.13 ± 0.03; n = 6; p = 0.001, compared with saline-injected control, Dunnett’s test) or by gain-up VOR training (0.22 ± 0.01; n = 6; p = 0.026) (Fig. 5; Supplementary Table S1), suggesting that activation of α2-adrenergic receptor counteracted the increased β-adrenergic receptor activity that resulted from OKR training or gain-up VOR training. α2-adrenergic agonist yohimbine did not significantly affect gain changes of OKR or VOR by any training (Figs 5 and 6, Supplementary Tables S1, S2).

### Effects of norepinephrine on VOR and OKR

**Figure 7:**
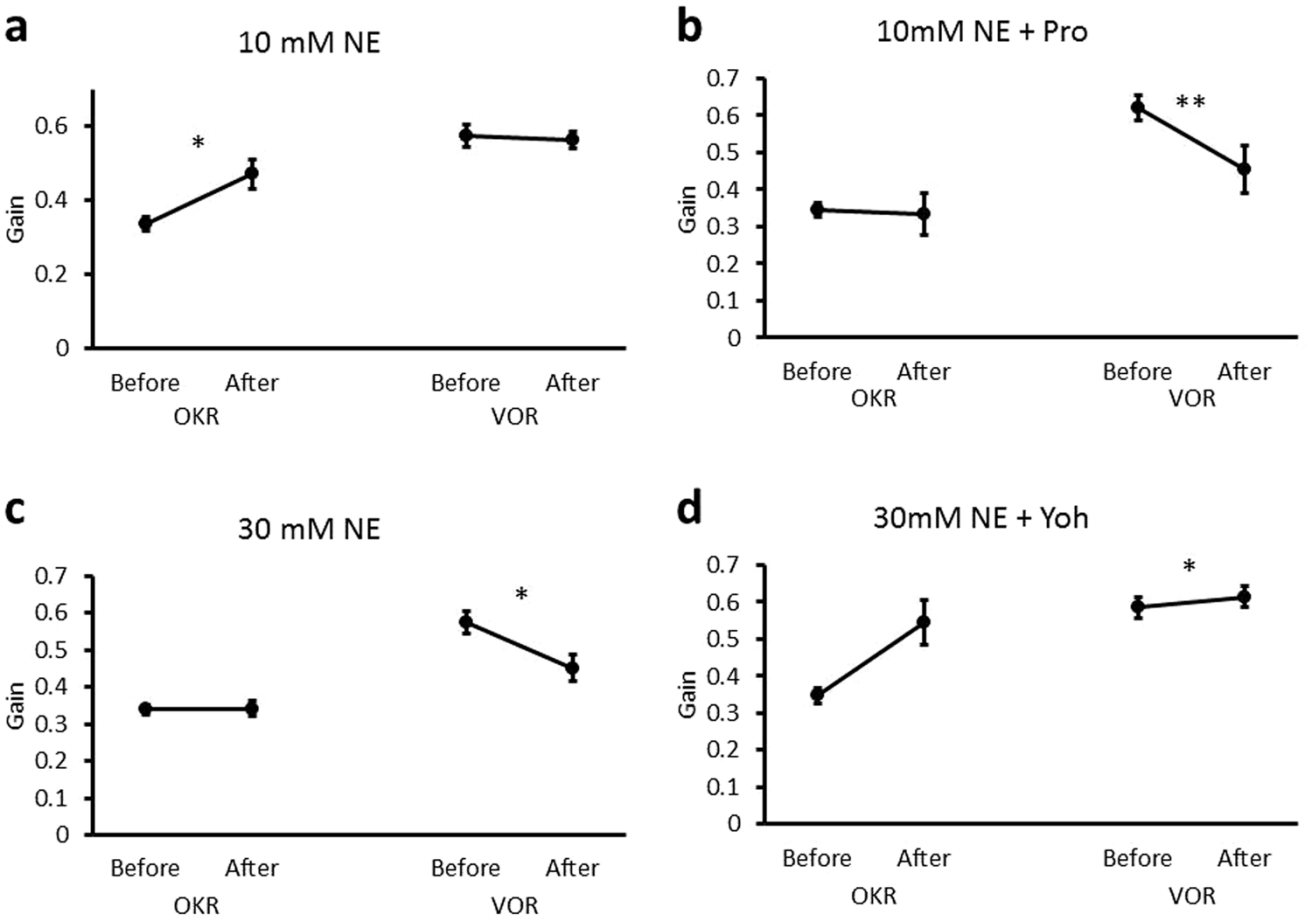
Effects of norepinephrine application on the OKR and VOR gains. (**a,b**), Effects of 10 mM norepinephrine (NE) without (**a**) or with propranolol (Pro) (**b**). (**c,d**) Effects of 30 mM norepinephrine without (**c**) or with yohimbine (Yoh) (**d**). Error bars indicate SEM. *p < 0.05, **p < 0.01, paired Student’s t-test.

Finally, we examined the effects of an endogenous agonist of adrenergic receptors norepinephrine on OKR and VOR. Application of 10 mM norepinephrine increased the OKR gain (before, 0.34 ± 0.02; after, 0.47 ± 0.04; n = 6; p = 0.049) but not the VOR gain (before, 0.57 ± 0.03; after, 0.56 ± 0.02; n = 6; p = 0.57) (Fig. 7, Supplementary Tables S10, S11), similarly to application of isoproterenol (Figs 1, 5 and 6; Supplementary Tables S1, S2). The increase of OKR gain was suppressed by co-application of propranolol (before, 0.34 ± 0.02; after, 0.33 ± 0.06; n = 6; p = 0.85) (Fig. 7; Supplementary Table S10). In contrast, this co-application decreased the VOR gain (before, 0.62 ± 0.03; after, 0.45 ± 0.06; n = 6; p = 0.009), similarly to application of propranolol alone (Fig. 7; Supplementary Tables S2, S11).

Application of 30 mM norepinephrine decreased the VOR gain (before, 0.58 ± 0.03; after, 0.45 ± 0.03; n = 6; p = 0.024) (Fig. 7; Supplementary Table S11) similarly to application of UK14304 (Fig. 5; Supplementary Table S2). 30 mM Norepinephrine did not influence the OKR gain, but additional application of yohimbine increased the OKR gain (before, 0.35 ± 0.02; after, 0.54 ± 0.06; n = 6; p = 0.026) similarly to application of 10 mM norepinephrine or isoproterenol (Supplementary Tables S1, S10). These results suggest that norepinephrine predominantly activates β-adrenergic receptor at a low concentration, whereas at a high concentration norepinephrine additionally activates α2-adrenergic receptor. Significant effects of norepinephrine on the phases of OKR and VOR were not detected (Supplementary Tables S12, S13).

## Discussion

Here, we showed that administration of β-adrenergic agonist into the flocculus increased the OKR gain but not the VOR gain, whereas administration there of β-adrenergic antagonist or α2-adrenergic agonist decreased the VOR gain and suppressed the gain-up adaptation of OKR. These results suggest that β-adrenergic receptor contributes to adaptation of OKR and keeping the VOR gain at a high level in a naive condition, and that α2-adrenergic receptor counteracts the β-receptor activity.

We also showed that administration of norepinephrine at 10 mM increased the OKR gain, whereas at 30 mM it decreased the VOR gain and had no effect on the OKR gain. These effects were suppressed by β- or α2-adrenergic antagonist, respectively. We think that endogenous norepinephrine binds to a substantial amount of β-receptor involved in VOR regulation in the basal condition, so that additional increase of β-adrenergic agonist such as norepinephrine or isoproterenol does not enhance the VOR gain because of a saturation effect of binding, although there could be other possibilities. We also suppose that α2-adrenergic receptor activation by norepinephrine at a high concentration or by UK143043 counteracted effects of β-adrenergic receptor activation through suppression of adenylyl cyclase. Thus, we conclude that norepinephrine predominantly activates β-receptor at a low concentration, and activates α2-receptor only at a high concentration in the flocculus. These results are in line with a previous study on cerebellar slice preparations, in which norepinephrine increased the firing frequency of Purkinje cells at low concentrations through β-receptor and decreased it at high concentrations through α-receptor^26^.

Our results showing that activation of β-adrenergic receptor increased the OKR gain, that β-adrenergic antagonist suppressed the gain increase of OKR by OKR training, gain-up or gain-down VOR training, and that a low concentration of norepinephrine predominantly activated β-adrenergic receptor, together suggest that norepinephrine-dependent activation of β-adrenergic receptor in the flocculus is involved in the OKR gain increase caused by various types of training.

On the other hand, our finding that β-adrenergic antagonists did not affect the gain-up VOR adaptation is contradictory to a previous report that found suppression of the gain-up VOR adaptation by β-adrenergic antagonist sotalol in rabbits^20^. The effects of β-adrenergic antagonist on the basal VOR gain were also different between the two studies. We found that propranolol or timolol decreased the VOR gain, whereas the previous study found no effect of sotalol. Differences in antagonists, animal species or other experimental conditions could be the cause of these differences. The norepinephrine concentration or expression patterns of adrenergic receptors in the flocculus might be different between mice and rabbits. We also note that sotalol inhibits not only β-adrenergic receptors but also potassium channels^27^.

In this study we focused on the flocculus among the floccular complex including the paraflocculus, because some studies reported that the flocculus played more critical role in regulation of OKR and VOR than the paraflocculus^4, 28^. We detected significant effects of drug injection to the flocculus on OKR and VOR as described above. However, the injected drug often diffused into the paraflocculus, and we do not deny possible contribution of the paraflocculus to regulation of OKR and VOR.

Asymmetrical regulation of eye movement during constant-velocity optokinetic stimulation was reported recently^23^. Thus, we examined effects of isoproterenol and propranolol on TN and NT eye movement during 2 sec 10 °/sec constant-velocity optokinetic stimulation. TN and NT eye movements were enhanced by isoproterenol application and unaffected by propranolol application. We did not detect significant asymmetrical effects of isoproterenol between the two directions. On the other hand, we detected asymmetrical regulation of eye velocity during the 2 sec constant-velocity optokinetic stimulation; the NT eye movement got faster, whereas the TN movement kept constant velocity. These results together with previous report^23^ suggest that neuronal circuits regulating OKR works asymmetrically. Thorough future analyses of several OKR parameters in various stimulation conditions such as different stimulation velocity, duration, with turning off and on of the light, or during OKR training might reveal asymmetrical effects of adrenergic drugs on OKR.

We do not know what kind of neuronal activity changes induced by activation of β-adrenergic receptor mediates the OKR gain increase. There are several subtypes in both β- and α2-adrenergic receptors^22^, and they are widely expressed in the cerebellar cortex^15, 29, 30^, although the precise sub-cellular localization of each subtype is unclear. β1-adrenergic receptor showing high affinity to norepinephrine is predominantly expressed in Purkinje neuron, whereas low affinity β2-adrenerigic receptor is expressed in other types of cerebellar cortical neurons^15^. All types of cerebellar cortical neurons express α2a- and α2b-adrenergic receptors in human^30^.

Previous studies showed that activation of β-adrenergic receptor enhances the Purkinje neuron activity^26^, parallel fiber synaptic or glutamate responses of Purkinje neurons^31, 32^, and inhibitory synaptic or GABA responses of Purkinje neurons^33, 34^, and that α2-adrenergic activity decreases the Purkinje neuron activity^26^, and parallel fiber or inhibitory synaptic responses of Purkinje neurons^32, 35^. It has also been reported that stimulation of the locus coeruleus enhances the Purkinje neuron response to climbing fiber activation^14^, and that α2-adrenergic receptor activity decreases the glutamate release from climbing fibers^36^. Whether any of these effects are directly related to the OKR gain increase or the maintenance of high basal VOR gain is unclear.

Synaptic plasticity in the cerebellum has been reported to contribute to the regulations of VOR and OKR. In particular, involvement of LTD at parallel fiber – Purkinje cell synapses induced by coupled activation of parallel fibers and a climbing fiber has been intensively studied^1, 7, 9^, although normal OKR and VOR adaptations in mutant mice defective in LTD were also reported^37^. In addition, climbing fiber activity-dependent enhancement of inhibitory synaptic transmission onto a Purkinje cell was shown to contribute to VOR adaptation^8^. There are several synaptic or intrinsic plasticity mechanisms in the cerebellum that could support motor learning^9, 38–40^.

The above plasticity mechanisms might be regulated by β- or α2-adrenergic receptor activity through Gs or Gi protein controlling intracellular cAMP concentration. cAMP facilitates RP induction^41–43^, and molecular signaling cascades regulating LTD could also be influenced by cAMP in a Purkinje cell, although cGMP plays a more important role in LTD induction^44^. The cAMP concentration-dependence might differ between LTD and RP. It was shown that the intracellular Ca^2+^ threshold for LTD induction is lower than that for RP induction^45^. Different contributions of LTD and RP to VOR and OKR adaptation have also been reported. In delphilin knockout mice, LTD is more easily induced than in wild-type mice and OKR adaptation is facilitated^10^, whereas VOR adaptation is not^11^. In RP-deficient transgenic mice, VOR adaptation is suppressed but OKR adaptation is not^8^. These results raise the possibility that the intra-Purkinje cell cAMP level regulated by β- and α2-adrenergic receptor controls LTD and RP differently, and also affects VOR and OKR differently. Other plasticity mechanisms such as intrinsic excitability plasticity^46^ could be also affected by cAMP and might affect VOR and OKR differently.

The visual, vestibular, and efference-copy or eye movement-related parallel fiber inputs to Purkinje cells in the flocculus are likely to show different firing patterns during OKR and VOR training, and might undergo different activity-dependent plasticity such as LTD and/or RP in the presence of norepinephrine. The present finding that OKR training in the presence of β-adrenergic agonist increased VOR gain might be explained by a neuronal activity-dependent plasticity mechanism facilitated by activation of β-adrenergic receptor.

There are still other possible mechanisms that could differentially modulate OKR and VOR by norepinephrine. For example, the visual, vestibular, and efference-copy or eye movement-related mossy fiber inputs to granule cells might have different sensitivity to norepinephrine. It has also been reported that there are several types of Purkinje cells showing different firing patterns during OKR and VOR^37^, and Purkinje cells with different output patterns might have different norepinephrine sensitivity. These possibilities are not mutually exclusive, and detailed future studies are necessary to clarify how the noradrenergic system in the flocculus modulates OKR and VOR differently.

## Methods

We used 8–10 weeks old male C57BL/6 mice for VOR and OKR recordings, and experimental procedures were carried out in accordance with the guidelines laid down by the National Institutes of Health of the USA and by Kyoto University, and approved by the local committee for handling experimental animals in the Graduate School of Science, Kyoto University.

The methods of eye movement recording and injection of drugs into the cerebellar flocculus were similar to those described previously^8, 10, 47, 48^. Briefly, a mouse was anesthetized by intraperitoneal injection of a mixture of 0.9% ketamine and 0.2% xylazine. A head holder was fixed to the top of the skull with small screws and dental cement. The next day, under the same anesthesia protocol as described above, craniotomy of about 1 mm diameter was made on the left periotic capsule, and a Teflon cannula was placed into the hole aiming at the flocculus and then affixed with dental acrylic. After this surgery, the mouse was allowed to recover for at least 48 hours.

For recording OKR or VOR, a mouse was fixed on a turntable surrounded by a cylindrical screen (diameter 30 cm, custom made) with vertical black and white stripes (14°). Sinusoidal oscillation of the turntable in the dark or that of the surrounding screen in the light at 1 Hz and 10°/sec peak velocity, was applied to induce VOR or OKR, respectively, unless otherwise stated. The right eye movement was monitored with an infrared-sensitive CCD camera (XC-HR50, Sony, Japan) under illumination by an infrared LED^47^. The image was sampled at 200 Hz, and the center of the pupil was calculated to estimate the eye position using the software (Geteye, Morita, Kyoto, Japan). A drop of pilocarpine hydrochloride (Santen, Osaka, Japan) was used to decrease and stabilize the pupil size during VOR recording in the dark. OKR or VOR were recorded for 30 seconds, 3 times with intervals of about 30 seconds in a mouse.

OKR or VOR performance was evaluated by measuring gain and phase as in previous studies^8, 10^. Eye position traces without rapid eye movement or eye blinking during each 30 second recording period was used to estimate the gain and phase of OKR or VOR. First, eye velocity was calculated as the difference between consecutive eye position values divided by the sampling interval of 5 msec. Then, at least 10 cycles of eye velocity curve were averaged and fitted with a sinusoidal curve by a least square method. The gain was defined as the amplitude of fitted sinusoidal eye velocity curve divided by that of the velocity curve of screen or turntable. The phase lag of eye velocity was defined as positive, and the phase zero for VOR was defined as the condition in which the head and eye movements were 180° out of phase. Thus, the phase zero corresponds to the ideal condition for stabilizing the visual image both in OKR and VOR. Estimated values from 3 trials were averaged in each mouse. N indicates the number of mice examined in each experiment, and the mean ± standard error of the measurement is presented.

Asymmetrical eye movement was examined by applying constant velocity optokinetic stimulation. The screen was rotated at 10°/sec for 2 sec in one direction. Then, the direction of screen rotation was reversed by linearly changing the rotation speed for 1 sec. Alternation of the rotation direction was repeated for 30 times. More than 9 eye position traces without rapid eye movement were averaged. The mean eye velocity at each time was calculated from mean eye positions.

For OKR or VOR training, a cycle consisting of 50 sec training followed by 10 sec resting period was repeated for 60 minutes in the light. The OKR training consisted of sinusoidal screen rotation at 1 Hz, 10°/sec peak velocity. The gain-up VOR training consisted of sinusoidal turntable rotation at 1 Hz, 10°/sec peak velocity coupled with out-of-phase rotation of the screen at 5°/sec peak velocity. The gain-down VOR training consisted of sinusoidal turntable rotation at 1 Hz, 10°/sec peak velocity coupled with in-phase rotation of the screen at 1 Hz, 10°/sec peak velocity. 0.6 μl of agonist or antagonist for adrenergic receptors dissolved in saline (76 mM isoproterenol, 100 mM propranolol, 10 mM yohimbine, 10 or 30 mM norepinephrine, 1 mM UK14304 containing 10% DMSO, all from Sigma-Aldrich, St. Louis, USA; 14 mM salbutamol, Tocris, Bristol, UK; 20 mM timolol, Wako, Osaka, Japan)^29, 49–51^ was applied to the left flocculus through a teflon cannula using a micro-syringe (701 N, Hamilton, Reno, USA). Isoproterenol or propranolol was injected into the right flocculus in some experiments. In some cases, the location of drug administration was examined by injecting a fluorescent dye (Lucifer yellow (0.1%, Sigma-Aldrich) or acriflavine hydrochloride (0.01%, Wako)) followed by observation of cerebellar slices.

The effects of agonist or antagonist on VOR or OKR were examined one day after recording VOR or OKR in naive untreated mice that had not experienced drug administration or training except for asymmetrical OKR examination, in which eye movement was recorded before and after drug administration on a same day with an interval of >3 hours. To study effects of agonist or antagonist on OKR or VOR adaptation, each drug was applied at the start of the first training cycle. The two-tailed paired Student’s t-test was used to compare mean values of gain and phase of OKR or VOR between before and after each treatment, after checking the normality of data with the Kolmogorov-Smirnov test. Dunnett’s test was used to compare mean values of gain and phase in each experimental group with those obtained from a corresponding saline-injected control group. We set the α level at 0.05.

## Electronic supplementary material

Supplementary Tables
