# Supplementary material for: Differential regulations of vestibulo-ocular reflex and optokinetic response by β- and α2-adrenergic receptors in the cerebellar flocculus
Source: Sci Rep. 2017 Jun 21;7:3944. doi: 10.1038/s41598-017-04273-9 (PMC5479797; doi:10.1038/s41598-017-04273-9)
Supplement: Supplementary file 1 — Supplementary Tables [file 41598_2017_4273_MOESM1_ESM.pdf]

Differential regulations of vestibulo-ocular reflex and optokinetic response by β- and α2-adrenergic receptors in the cerebellar flocculus

Ryo Wakita[1,] Soshi Tanabe[1], Kazunari Tabei[1], Asako Funaki[1], Takuma Inoshita[1], Tomoo Hirano[1]

[1]Department of Biophysics, Graduate School of Science, Kyoto University, Sakyo-ku, Kyoto 606-8502, Japan

Supplementary Table S1

Effects of various treatments on OKR gain at 1 Hz, 10°/sec peak screen rotation velocity

| Drug | Side | Training | Pre | Post | Change | N | T-test | Dunnett |
|---|---|---|---|---|---|---|---|---|
| saline | contra | no | 0.31±0.02 | 0.31±0.03 | 0.01±0.03 | 6 | 0.813 | |
| | | OKR-up | 0.29±0.02 | 0.66±0.03 | 0.37±0.03 | 6 | <0.001 | |
| | | VOR-up | 0.33±0.02 | 0.69±0.05 | 0.36±0.04 | 6 | <0.001 | |
| | | VOR-down | 0.31±0.02 | 0.52±0.03 | 0.21±0.03 | 6 | 0.001 | |
| | ipsi | no | 0.31±0.01 | 0.31±0.02 | 0.00±0.02 | 6 | 0.870 | |
| isoproterenol | contra | no | 0.33±0.02 | 0.47±0.02 | 0.14±0.02 | 6 | <0.001 | 0.012 |
| | | OKR-up | 0.35±0.02 | 0.64±0.05 | 0.29±0.05 | 6 | 0.002 | 0.388 |
| | | VOR-up | 0.31±0.02 | 0.69±0.05 | 0.38±0.05 | 6 | 0.001 | 0.981 |
| | | VOR-down | 0.33±0.03 | 0.63±0.03 | 0.30±0.03 | 6 | <0.001 | 0.154 |
| | ipsi | no | 0.31±0.02 | 0.44±0.03 | 0.13±0.03 | 6 | 0.005 | 0.002 |
| propranolol | contra | no | 0.32±0.02 | 0.28±0.03 | −0.04±0.02 | 6 | 0.111 | 0.732 |
| | | OKR-up | 0.35±0.02 | 0.42±0.03 | 0.07±0.03 | 6 | 0.095 | < 0.001 |
| | | VOR-up | 0.31±0.02 | 0.36±0.02 | 0.05±0.02 | 6 | 0.105 | < 0.001 |
| | | VOR-down | 0.29±0.03 | 0.36±0.02 | 0.07±0.02 | 6 | 0.045 | 0.016 |
| | ipsi | no | 0.27±0.02 | 0.27±0.01 | 0.00±0.02 | 7 | 0.985 | 0.994 |
| UK14304 | contra | no | 0.32±0.02 | 0.27±0.02 | −0.05±0.02 | 6 | 0.066 | 0.532 |
| | | OKR-up | 0.33±0.02 | 0.46±0.04 | 0.13±0.04 | 6 | 0.017 | 0.001 |
| | | VOR-up | 0.30±0.02 | 0.52±0.02 | 0.22±0.01 | 6 | <0.001 | 0.026 |
| | | VOR-down | 0.28±0.02 | 0.44±0.04 | 0.16±0.03 | 6 | 0.005 | 0.697 |
| yohimbine | contra | no | 0.30±0.02 | 0.42±0.03 | 0.12±0.03 | 7 | 0.010 | 0.034 |
| | | OKR-up | 0.30±0.03 | 0.61±0.06 | 0.31±0.05 | 7 | <0.001 | 0.607 |
| | | VOR-up | 0.28±0.01 | 0.57±0.03 | 0.29±0.02 | 6 | <0.001 | 0.419 |
| | | VOR-down | 0.28±0.02 | 0.47±0.04 | 0.19±0.04 | 6 | 0.008 | 0.973 |
| salbutamol | contra | no | 0.35±0.05 | 0.51±0.07 | 0.15±0.04 | 6 | 0.008 | 0.006 |
| timolol | contra | no | 0.37±0.03 | 0.33±0.03 | −0.04±0.03 | 7 | 0.234 | 0.628 |
| DMSO | contra | no | 0.32±0.03 | 0.34±0.02 | 0.02±0.02 | 6 | 0.494 | 1.000 |

Administration of saline, isoproterenol, propranolol, UK14304, yohimbine, salbutamol, timolol, or DMSO to the flocculus contralateral or ipsilateral to the recorded eye, with or without OKR, VOR-up or VOR-down training was performed. The effect of each treatment on OKR gain was examined between pre- and post-treatment with the paired t-test. The effect of each drug on OKR gain change in each training condition was compared with that of saline-administered control with Dunnett's test. P values are presented.

Supplementary Table S2

Effects of various treatment on VOR gain at 1 Hz, 10°/sec peak turntable rotation velocity

| Drug | Side | Training | Pre | Post | Change | N | T-test | Dunnett |
|---|---|---|---|---|---|---|---|---|
| saline | contra | no | 0.61±0.02 | 0.63±0.02 | 0.01±0.02 | 6 | 0.564 | |
| | | OKR-up | 0.59±00.2 | 0.60±0.03 | 0.01±0.03 | 6 | 0.637 | |
| | | VOR-up | 0.62±0.02 | 0.81±0.05 | 0.19±0.05 | 6 | 0.011 | |
| | | VOR-down | 0.61±0.02 | 0.41±0.02 | −0.20±0.02 | 6 | <0.001 | |
| | ipsi | no | 0.71±0.02 | 0.72±0.04 | 0.01±0.03 | 6 | 0.821 | |
| isoproterenol | contra | no | 0.66±0.01 | 0.63±0.04 | −0.04±0.04 | 6 | 0.415 | 0.695 |
| | | OKR-up | 0.64±0.04 | 0.81±0.03 | 0.17±0.03 | 6 | 0.004 | 0.016 |
| | | VOR-up | 0.66±0.04 | 0.82±0.05 | 0.16±0.04 | 6 | 0.013 | 0.959 |
| | | VOR-down | 0.61±0.03 | 0.40±0.03 | −0.21±0.03 | 6 | 0.002 | 0.999 |
| | ipsi | no | 0.72±0.04 | 0.77±0.04 | 0.05±0.06 | 6 | 0.441 | 0.717 |
| propranolol | contra | no | 0.64±0.03 | 0.51±0.02 | −0.13±0.04 | 6 | 0.017 | 0.010 |
| | | OKR-up | 0.62±0.03 | 0.52±0.04 | −0.10±0.06 | 6 | 0.154 | 0.091 |
| | | VOR-up | 0.58±0.04 | 0.71±0.04 | 0.13±0.04 | 6 | 0.029 | 0.768 |
| | | VOR-down | 0.69±0.03 | 0.43±0.04 | −0.26±0.03 | 6 | <0.001 | 0.470 |
| | ipsi | no | 0.74±0.06 | 0.58±0.06 | −0.15±0.04 | 7 | 0.005 | 0.031 |
| UK14304 | contra | no | 0.65±0.04 | 0.49±0.04 | −0.16±0.03 | 6 | 0.004 | 0.001 |
| | | OKR-up | 0.65±0.02 | 0.65±0.03 | 0.00±0.01 | 6 | 0.949 | 0.995 |
| | | VOR-up | 0.61±0.03 | 0.74±0.05 | 0.13±0.06 | 6 | 0.063 | 0.792 |
| | | VOR-down | 0.62±0.03 | 0.49±0.04 | −0.13±0.03 | 6 | 0.010 | 0.328 |
| yohimbine | contra | no | 0.65±0.04 | 0.69±0.04 | 0.04±0.02 | 7 | 0.053 | 0.990 |
| | | OKR-up | 0.68±0.03 | 0.73±0.04 | 0.05±0.02 | 7 | 0.091 | 0.901 |
| | | VOR-up | 0.64±0.04 | 0.85±0.03 | 0.21±0.04 | 6 | 0.002 | 0.994 |
| | | VOR-down | 0.63±0.03 | 0.38±0.02 | −0.25±0.03 | 6 | <0.001 | 0.631 |
| salbutamol | contra | no | 0.52±0.05 | 0.56±0.05 | 0.04±0.02 | 6 | 0.085 | 0.993 |
| timolol | contra | no | 0.65±0.04 | 0.43±0.04 | −0.23±0.04 | 7 | 0.001 | < 0.001 |
| DMSO | contra | no | 0.58±0.04 | 0.61±0.04 | 0.02±0.01 | 6 | 0.161 | 1.000 |

Administration of saline, isoproterenol, propranolol, UK14304, yohimbine, salbutamol, timolol, or DMSO to the flocculus contralateral or ipsilateral to the recorded eye, with or without OKR, VOR-up or VOR-down training was performed. The effect of each treatment on VOR gain was examined between pre- and post-treatment with the paired t-test. The effect of each drug on VOR gain change in each training condition was compared with that of saline-administered control with Dunnett's test. P values are presented.

Supplementary Table S3

Effects of contralateral administration of propranolol or isoproterenol on OKR gain during screen rotation (0.5 Hz, 10°/sec peak rotation velocity; 1 Hz, 20°/sec)

| Drug | Frequency | Peak velocity | Pre | Post | Change | N | T-test | Dunnett |
|---|---|---|---|---|---|---|---|---|
| saline | 0.5 | 10 | 0.41±0.04 | 0.41±0.05 | 0.00±0.03 | 6 | 0.943 | |
| | 1 | 20 | 0.17±0.02 | 0.17±0.02 | 0.00±0.01 | 6 | 0.679 | |
| isoproterenol | 0.5 | 10 | 0.45±0.03 | 0.60±0.04 | 0.15±0.06 | 6 | 0.040 | 0.017 |
| | 1 | 20 | 0.17±0.01 | 0.21±0.02 | 0.04±0.01 | 6 | 0.042 | 0.044 |
| propranolol | 0.5 | 10 | 0.35±0.03 | 0.34±0.04 | -0.02±0.02 | 6 | 0.380 | 0.946 |
| | 1 | 20 | 0.18±0.02 | 0.20±0.02 | 0.02±0.01 | 6 | 0.095 | 0.432 |

The effect of drug application on OKR gain was compared between before and after the administration with the paired t-test in each condition. The drug effect on OKR gain was also compared with that of saline-administered control with Dunnett's test in each rotation condition. P values are presented.

Supplementary Table S4

Effects of contralateral administration of propranolol or isoproterenol on VOR gain during turntable rotation (0.5 Hz, 10°/sec peak rotation velocity; 1 Hz, 20°/sec)

| Drug | Frequency | Peak velocity | Pre | Post | Change | N | T-test | Dunnett |
|---|---|---|---|---|---|---|---|---|
| saline | 0.5 | 10 | 0.29±0.03 | 0.28±0.03 | −0.01±0.01 | 6 | 0.439 | |
| | 1 | 20 | 0.60±0.06 | 0.62±0.03 | 0.02±0.05 | 6 | 0.752 | |
| isoproterenol | 0.5 | 10 | 0.36±0.03 | 0.28±0.03 | −0.08±0.04 | 6 | 0.114 | 0.118 |
| | 1 | 20 | 0.65±0.03 | 0.61±0.05 | −0.04±0.04 | 6 | 0.407 | 0.568 |
| propranolol | 0.5 | 10 | 0.37±0.03 | 0.28±0.03 | −0.10±0.01 | 6 | <0.001 | 0.041 |
| | 1 | 20 | 0.66±0.03 | 0.49±0.05 | −0.17±0.03 | 6 | 0.003 | 0.014 |

The effect of drug application on VOR gain was compared between before and after the administration with the paired t-test in each condition. The drug effect on VOR gain was also compared with that of saline-administered control with Dunnett's test in each rotation condition. P values are presented.

Supplementary Table S5

Effects of various treatments on OKR phase at 1 Hz, 10°/sec peak screen rotation velocity

| Drug | Side | Training | Pre | Post | Change | N | T-test | Dunnett |
|---|---|---|---|---|---|---|---|---|
| saline | contra | no | 29±2 | 29±1 | 0±3 | 6 | 0.977 | |
| | | OKR-up | 27±3 | 18±1 | -9±3 | 6 | 0.017 | |
| | | VOR-up | 31±2 | 20±1 | -11±2 | 6 | 0.002 | |
| | | VOR-down | 28±1 | 13±2 | -14±2 | 6 | 0.002 | |
| | ipsi | no | 34±3 | 37±2 | 3±2 | 6 | 0.163 | |
| isoproterenol | contra | no | 28±2 | 21±1 | -7±1 | 6 | 0.004 | 0.683 |
| | | OKR-up | 26±3 | 15±2 | -11±3 | 6 | 0.024 | 0.906 |
| | | VOR-up | 29±2 | 23±2 | -5±3 | 6 | 0.120 | 0.260 |
| | | VOR-down | 28±2 | 17±1 | -12±2 | 6 | 0.001 | 0.903 |
| | ipsi | no | 38±1 | 30±1 | -8±2 | 6 | 0.007 | 0.021 |
| propranolol | contra | no | 28±1 | 31±3 | 3±3 | 6 | 0.469 | 0.996 |
| | | OKR-up | 29±1 | 20±2 | -9±1 | 6 | <0.001 | 1.000 |
| | | VOR-up | 29±2 | 26±3 | -3±2 | 6 | 0.110 | 0.087 |
| | | VOR-down | 28±3 | 13±1 | -15±4 | 6 | 0.013 | 1.000 |
| | ipsi | no | 34±3 | 39±5 | 5±3 | 7 | 0.187 | 0.837 |
| UK14304 | contra | no | 27±1 | 33±1 | 6±2 | 6 | 0.031 | 0.829 |
| | | OKR-up | 29±1 | 18±2 | -11±2 | 6 | 0.002 | 0.926 |
| | | VOR-up | 26±1 | 26±2 | 0±2 | 6 | 0.970 | 0.010 |
| | | VOR-down | 30±3 | 19±2 | -11±2 | 6 | 0.004 | 0.766 |
| yohimbine | contra | no | 30±1 | 28±2 | -2±2 | 7 | 0.347 | 1.000 |
| | | OKR-up | 28±1 | 15±1 | -13±2 | 7 | <0.001 | 0.515 |
| | | VOR-up | 26±2 | 23±1 | -3±3 | 6 | 0.371 | 0.071 |
| | | VOR-down | 28±2 | 17±3 | -11±3 | 6 | 0.013 | 0.878 |
| salbutamol | contra | no | 28±2 | 21±2 | -7±3 | 6 | 0.077 | 0.675 |
| timolol | contra | no | 29±2 | 41±8 | 12±8 | 7 | 0.175 | 0.133 |
| DMSO | contra | no | 34±2 | 31±1 | -3±2 | 6 | 0.200 | 0.994 |

Administration of saline, isoproterenol, propranolol, UK14304, yohimbine, salbutamol, timolol, or DMSO to the flocculus contralateral or ipsilateral to the recorded eye, with or without OKR, VOR-up or VOR-down training was performed. The effect of each treatment on OKR phase was examined between pre- and post-treatment with the paired t-test. The effect of each drug on OKR phase change in each training condition was compared with that of saline-administered control with Dunnett's test. P values are presented. A positive phase value indicates a delay of eye rotation relative to the screen rotation.

Supplementary Table S6

Effects of various treatments on VOR phase at 1 Hz, 10°/sec peak turntable rotation velocity

| Drug | Side | Training | Pre | Post | Change | N | T-test | Dunnett |
|---|---|---|---|---|---|---|---|---|
| saline | contra | no | −29±2 | −27±3 | 2±3 | 6 | 0.535 | |
| | | OKR-up | −22±2 | −28±1 | −7±2 | 6 | 0.034 | |
| | | VOR-up | −20±1 | −18±2 | 1±1 | 6 | 0.471 | |
| | | VOR-down | −18±2 | −33±4 | −15±3 | 6 | 0.006 | |
| | ipsi | no | −23±3 | −24±2 | −1±3 | 6 | 0.776 | |
| isoproterenol | contra | no | −22±2 | −34±3 | −11±4 | 6 | 0.035 | 0.217 |
| | | OKR-up | −27±4 | −31±2 | −4±3 | 6 | 0.255 | 0.749 |
| | | VOR-up | −28±2 | −29±4 | −1±5 | 6 | 0.822 | 0.946 |
| | | VOR-down | −22±3 | −35±4 | −12±3 | 6 | 0.010 | 0.939 |
| | ipsi | no | −25±4 | −31±3 | −7±3 | 6 | 0.112 | 0.328 |
| propranolol | contra | no | −19±1 | −17±1 | 2±1 | 6 | 0.155 | 1.000 |
| | | OKR-up | −25±2 | −27±3 | −2±2 | 6 | 0.494 | 0.372 |
| | | VOR-up | −24±3 | −22±1 | 2±3 | 6 | 0.503 | 0.999 |
| | | VOR-down | −24±3 | −26±2 | −1±2 | 6 | 0.567 | 0.020 |
| | ipsi | no | −20±3 | −13±3 | 8±3 | 7 | 0.032 | 0.107 |
| UK14304 | contra | no | −22±1 | −20±2 | 2±2 | 6 | 0.412 | 1.000 |
| | | OKR-up | −25±2 | −26±2 | −1±2 | 6 | 0.634 | 0.254 |
| | | VOR-up | −22±2 | −19±2 | 3±1 | 6 | 0.054 | 0.982 |
| | | VOR-down | −21±3 | −27±2 | −6±3 | 6 | 0.070 | 0.170 |
| yohimbine | contra | no | −18±1 | −24±2 | −6±2 | 7 | 0.037 | 0.692 |
| | | OKR-up | −17±2 | −22±2 | −5±2 | 7 | 0.047 | 0.926 |
| | | VOR-up | −24±1 | −18±2 | 6±3 | 6 | 0.071 | 0.593 |
| | | VOR-down | −20±1 | −26±5 | −6±4 | 6 | 0.214 | 0.175 |
| salbutamol | contra | no | −20±4 | −48±9 | −28±12 | 6 | 0.063 | < 0.001 |
| timolol | contra | no | −27±2 | −22±3 | 5±2 | 7 | 0.062 | 0.998 |
| DMSO | contra | no | −23±2 | −19±2 | 5±1 | 6 | 0.026 | 0.999 |

Administration of saline, isoproterenol, propranolol, UK14304, yohimbine, salbutamol, timolol, or DMSO to the flocculus contralateral or ipsilateral to the recorded eye, with or without OKR, VOR-up or VOR-down training was performed. The effect of each treatment on VOR phase was examined between pre- and post-treatment with the paired t-test. The effect of each drug on VOR phase change in each training condition was compared with that of saline-administered control with Dunnett's test. P values are presented. A negative phase value indicates that the eye rotation occurs ahead of the screen rotation.

Supplementary Table S7

Effects of contralateral administration of propranolol or isoproterenol on OKR phase during screen rotation (0.5 Hz, 10°/sec peak rotation velocity; 1 Hz, 20°/sec)

| Drug | Frequency | Peak velocity | Pre | Post | Change | N | T-test | Dunnett |
|---|---|---|---|---|---|---|---|---|
| saline | 0.5 | 10 | 22±9 | 13±1 | -8±9 | 6 | 0.391 | |
| | 1 | 20 | 29±1 | 20±2 | -9±3 | 6 | 0.023 | |
| isoproterenol | 0.5 | 10 | 15±2 | 18±8 | 3±9 | 6 | 0.784 | 0.487 |
| | 1 | 20 | 28±3 | 30±7 | 2±10 | 6 | 0.851 | 0.361 |
| propranolol | 0.5 | 10 | 13±1 | 15±2 | 2±2 | 6 | 0.466 | 0.533 |
| | 1 | 20 | 30±1 | 27±3 | -3±3 | 6 | 0.401 | 0.709 |

The effect of drug application on OKR phase was compared between before and after the administration with the paired t-test in each condition. The drug effect on OKR phase was also compared with that of saline-administered control with Dunnett's test in each rotation condition. P values are presented. A positive phase value indicates a delay of eye rotation relative to the screen rotation.

Supplementary Table S8

Effects of contralateral administration of propranolol or isoproterenol on VOR phase during turntable rotation (0.5 Hz, 10°/sec peak rotation velocity; 1 Hz, 20°/sec)

| Drug | Frequency | Peak velocity | Pre | Post | Change | N | T-test | Dunnett |
|---|---|---|---|---|---|---|---|---|
| saline | 0.5 | 10 | −48±5 | −55±9 | −7±7 | 6 | 0.375 | |
| | 1 | 20 | −12±1 | −14±1 | −2±1 | 6 | 0.093 | |
| isoproterenol | 0.5 | 10 | −46±4 | −62±4 | −16±5 | 6 | 0.021 | 0.403 |
| | 1 | 20 | −13±2 | −28±4 | −14±5 | 6 | 0.032 | 0.015 |
| propranolol | 0.5 | 10 | −41±3 | −50±4 | −8±3 | 6 | 0.057 | 0.974 |
| | 1 | 20 | −16±1 | −19±1 | −4±1 | 6 | 0.037 | 0.831 |

The effect of drug application on VOR phase was compared between before and after the administration with the paired t-test in each condition. The drug's effect on VOR phase was also compared with that of saline-administered control with Dunnett's test in each rotation condition. P values are presented. A negative phase value indicates that the eye rotation occurs ahead of the screen rotation.

Supplementary Table S9

Effects of contralateral administration of propranolol or isoproterenol on OKR gain during constant-velocity optokinetic stimulation of NT or TN direction

| Drug | Period | Direction | Pre | Post | Change | N | T-test | Dunnett |
|---|---|---|---|---|---|---|---|---|
| saline | former | NT | 0.35±0.03 | 0.38±0.03 | 0.02±0.01 | 6 | 0.096 | |
| | | TN | 0.36±0.03 | 0.38±0.03 | 0.02±0.02 | 6 | 0.485 | |
| | | **T-test (NT vs TN)** | 0.584 | 0.895 | 0.217 | | | |
| | latter | NT | 0.48±0.03 | 0.46±0.03 | -0.01±0.02 | 6 | 0.374 | |
| | | TN | 0.39±0.03 | 0.38±0.03 | -0.01±0.02 | 6 | 0.930 | |
| | | **T-test (NT vs TN)** | 0.003 | 0.025 | 0.985 | | | |
| iso | former | NT | 0.30±0.02 | 0.53±0.05 | 0.23±0.05 | 6 | 0.004 | 0.001 |
| | | TN | 0.35±0.05 | 0.52±0.06 | 0.17±0.01 | 6 | <0.001 | 0.014 |
| | | **T-test (NT vs TN)** | 0.302 | 0.836 | 0.185 | | | |
| | latter | NT | 0.43±0.02 | 0.64±0.04 | 0.21±0.04 | 6 | 0.004 | <0.001 |
| | | TN | 0.34±0.04 | 0.48±0.06 | 0.14±0.03 | 6 | 0.005 | <0.001 |
| | | **T-test (NT vs TN)** | 0.006 | 0.032 | 0.216 | | | |
| pro | former | NT | 0.34±0.03 | 0.37±0.04 | 0.03±0.03 | 6 | 0.373 | 0.991 |
| | | TN | 0.34±0.03 | 0.42±0.05 | 0.08±0.05 | 6 | 0.142 | 0.356 |
| | | **T-test (NT vs TN)** | 0.997 | 0.151 | 0.245 | | | |
| | latter | NT | 0.43±0.03 | 0.44±0.04 | 0.01±0.02 | 6 | 0.763 | 0.943 |
| | | TN | 0.35±0.02 | 0.32±0.03 | -0.03±0.02 | 6 | 0.094 | 0.61 |
| | | **T-test (NT vs TN)** | 0.046 | 0.012 | 0.177 | | | |

Mean gain values during the former and the latter 1 sec periods of the 2 sec stimulation were presented. The effect of drug application on OKR gain was compared between before and after the administration with the paired t-test. The drug's effect on OKR gain was also compared with that of saline-administered control with Dunnett's test. In addition, the directional difference was examined with the paired t-test in each condition. P values are presented.

Supplementary Table S10

Effects of contralateral administration of norepinephrine (NE) on OKR gain

| NE | Additional drug | Pre | Post | Change | N | T-test | Dunnett |
|---|---|---|---|---|---|---|---|
| 10 mM | no | 0.34±0.02 | 0.47±0.04 | 0.14±0.05 | 6 | 0.049 | 0.204 |
| | propranolol | 0.34±0.02 | 0.33±0.06 | −0.01±0.05 | 6 | 0.852 | 0.996 |
| 30 mM | no | 0.34±0.01 | 0.34±0.02 | 0.00±0.02 | 6 | 0.937 | 1.000 |
| | yohimbine | 0.35±0.02 | 0.54±0.06 | 0.20±0.06 | 6 | 0.026 | 0.031 |

Administration of 10 mM NE with or without co-administration of propranolol, or that of 30 mM NE with or without co-administration of yohimbine was performed. The effect of each treatment on OKR gain was examined between before and after drug application with the paired t-test. The effect of each treatment on OKR gain change was also compared with that of saline-administered control with Dunnett's test. P values are presented.

Supplementary Table S11

Effects of contralateral administration of norepinephrine (NE) on VOR gain

| NE | Additional drug | Pre | Post | Change | N | T-test | Dunnett |
|---|---|---|---|---|---|---|---|
| 10 mM | no | 0.57±0.03 | 0.56±0.02 | −0.01±0.02 | 6 | 0.573 | 0.926 |
| | propranolol | 0.62±0.03 | 0.45±0.06 | −0.17±0.04 | 6 | 0.009 | < 0.001 |
| 30 mM | no | 0.58±0.03 | 0.45±0.03 | −0.12±0.04 | 6 | 0.024 | 0.008 |
| | yohimbine | 0.58±0.03 | 0.61±0.03 | 0.03±0.01 | 6 | 0.051 | 0.987 |

Administration of 10 mM NE with or without co-administration of propranolol, or that of 30 mM NE with or without co-administration of yohimbine was performed. The effect of each treatment on VOR gain was examined between before and after drug application with the paired t-test. The effect of each treatment on VOR gain change was also compared with that of saline-administered control with Dunnett's test. P values are presented.

Supplementary Table S12

Effects of contralateral administration of norepinephrine (NE) on OKR phase

| NE | Additional drug | Pre | Post | Change | N | T-test | Dunnett |
|---|---|---|---|---|---|---|---|
| 10 mM | no | 29±2 | 27±3 | −1±3 | 6 | 0.692 | 0.992 |
| | propranolol | 24±1 | 31±3 | 7±3 | 6 | 0.059 | 0.189 |
| 30 mM | no | 31±1 | 26±3 | −5±2 | 6 | 0.069 | 0.584 |
| | yohimbine | 27±2 | 22±2 | −5±3 | 6 | 0.091 | 0.458 |

Administration of 10 mM NE with or without co-administration of propranolol, or that of 30 mM NE with or without co-administration of yohimbine was performed. The effect of each treatment on OKR phase was examined between before and after drug application with the paired t-test. The effect of each treatment on OKR phase change was also compared with that of saline-administered control with Dunnett's test. P values are presented. A positive phase value indicates a delay of eye rotation relative to the screen rotation.

Supplementary Table S13
Effects of contralateral administration of norepinephrine (NE) on VOR phase

| NE | Additional drug | Pre | Post | Change | N | T-test | Dunnett |
|---|---|---|---|---|---|---|---|
| 10 mM | no | −28±3 | −30±5 | −2±4 | 6 | 0.640 | 0.932 |
| | propranolol | −23±3 | −27±5 | −3±6 | 6 | 0.580 | 0.858 |
| 30 mM | no | −20±1 | −22±2 | −2±2 | 6 | 0.452 | 0.951 |
| | yohimbine | −23±3 | −20±7 | 3±8 | 6 | 0.702 | 1.000 |

Administration of 10 mM NE with or without co-administration of propranolol, or that of 30 mM NE with or without co-administration of yohimbine, was performed. The effect of each treatment on VOR phase was examined between before and after drug application with the paired t-test. The effect of each treatment on VOR phase change was also compared with that of saline-administered control with Dunnett's test. P values are presented. A negative phase value indicates that the eye rotation occurs ahead of the screen rotation.
